# Clinical and Lifestyle Modulators of Cortisol and Folate Dysregulation in Schizophrenia: The Impact of Smoking, Thyroid Dysfunction, and Psychiatric History

**DOI:** 10.1192/bjo.2026.11125

**Published:** 2026-06-30

**Authors:** Najmeh Shahini, Mostafa Zare, Mahsa Omiddezyani, Abdurrahman Charkazi

**Affiliations:** 1 Birmingham and Solihull Mental Health Foundation Trust, Birmingham, United Kingdom; 2 Leicestershire Partnership NHS Trust, Leicester, United Kingdom; 3 Golestan Research Center of Psychiatry (GRCP), Golestan University of Medical Sciences, Gorgan,Iran, Islamic Republic of; 4 Psychotherapy Fellowship, Golestan Research Center of Psychiatry (GRCP), Golestan University of Medical Sciences, Gorgan, Iran, Islamic Republic of; 5 Health Research Center, Faculty of Health, Golestan University of Medical Sciences, Gorgan, Iran, Islamic Republic of

## Abstract

**Aims:**

Dysregulation of cortisol and folate–two biomarkers central to stress physiology and one-carbon metabolism–has been consistently reported in schizophrenia. However, the extent to which clinical and lifestyle variables shape these biomarkers remains insufficiently understood. Factors such as smoking, thyroid dysfunction, and prior psychiatric history are biologically relevant confounders: nicotine alters folate turnover and methylation pathways; hypothyroidism disrupts one-carbon metabolism and HPA-axis regulation; and long-standing psychiatric illness is associated with chronic stress-system alterations. These mechanisms may generate atypical biochemical signatures that complicate interpretation of peripheral biomarkers in schizophrenia. This study therefore investigated whether these variables contribute to unexpected cortisol and folate variations beyond the anticipated case–control differences.

**Methods:**

A case–control design was implemented involving 33 patients with schizophrenia (DSM–5 diagnosis confirmed by two consultant psychiatrists) and 33 age-and sex-matched healthy controls without personal or familial psychiatric history. To minimize circadian effects, fasting venous blood samples were collected between 07:00–08:00am. Serum cortisol and folate were measured using standardized AccuBind ELISA kits, with all assays performed in duplicate. Samples were processed under uniform laboratory conditions and stored at −20°C. Symptom severity and extrapyramidal side effects were assessed by a blinded psychiatrist using the PANSS and SAS scales. Demographic and clinical variables–including smoking status, thyroid disease, and psychiatric history–were systematically documented. Normality was assessed using the Shapiro–Wilk test; group comparisons employed t-tests or Mann–Whitney U tests; and associations were examined using Spearman correlations (p<0.05). Ethical approval was obtained from Golestan University of Medical Sciences.

**Results:**

Compared with healthy controls, patients with schizophrenia exhibited significantly lower serum cortisol (54.98 ± 26.90 vs. 96.10 ± 57.15 µg/dL; p<0.0001) and folate levels (2.21 ± 2.20 vs. 14.69 ± 11.68 ng/mL; p<0.0001). Beyond these expected findings, several distinct patterns emerged:

• Participants with a psychiatric history showed markedly reduced cortisol (12.16 ± 10.27 vs. 57.77 ± 26.45; p=0.017).

• Individuals with hypothyroidism demonstrated significantly lower folate (1.94 ± 1.89 vs. 5.30 ± 2.78; p=0.020).

• Smokers unexpectedly exhibited higher folate levels (4.70 ± 2.73 vs. 1.94 ± 1.86; p=0.036).

No significant correlations were found between biomarkers and PANSS or SAS scores.

**Conclusion:**

In addition to global reductions in cortisol and folate in schizophrenia, this study identifies distinct biomarker signatures linked to smoking, thyroid dysfunction, and psychiatric history. These findings underscore the importance of accounting for metabolic and lifestyle factors when interpreting peripheral biomarkers in psychiatric research and clinical practice.

